# Structural Insights into the Interaction Between a Core-Fucosylated Foodborne Hexasaccharide (H_2_N_2_F_2_) and Human Norovirus P Proteins

**DOI:** 10.3390/v18010131

**Published:** 2026-01-20

**Authors:** Zilei Zhang, Yuchen Wang, Jiaqi Xu, Fei Liu, Shumin Li, Justin Troy Cox, Liang Xue, Danlei Liu

**Affiliations:** 1 Department of Customs Inspection and Quarantine, Shanghai Customs University, Shanghai 201204, China; zhangzilei@shcc.edu.cn (Z.Z.);; 2 Department of Geratology of Minhang Hospital, Fudan University, Shanghai 221100, China; 3 Shandong Mental Health Center, Jinan 250014, China; 4 College of Veterinary Medicine, Jilin University, Changchun 130062, China; 5 Imagination Realty Network, LLC., Houston, TX 77040, USA; 6 State Key Laboratory of Applied Microbiology Southern China, Institute of Microbiology, Guangdong Academy of Sciences, Guangzhou 510070, China; 7 Shanghai International Travel Healthcare Center, Shanghai Customs District People’s Republic of China, Shanghai 200335, China

**Keywords:** norovirus, core-fucosylated hexasaccharide (H_2_N_2_F_2_), AlphaFold3, molecular docking, foodborne transmission

## Abstract

Background: Human noroviruses are the leading cause of foodborne gastroenteritis worldwide. Accumulating evidence suggests that food matrices containing fucosylated or histo-blood group antigen (HBGA)-like glycans may facilitate viral attachment and persistence, yet the molecular mechanisms underlying these interactions remain unclear. Methods: In this study, we performed a comparative computational analysis of norovirus–glycan interactions by integrating AlphaFold3-based structure prediction, molecular docking, and molecular dynamics simulations. A total of 182 P-domain models representing all genotypes across five human norovirus genogroups (GI, GII, GIV, GVIII, and GIX) were predicted and docked with a lettuce-derived core-fucosylated hexasaccharide (H_2_N_2_F_2_) previously identified by our group. The three complexes exhibiting the most favorable docking energies were further examined using 40 ns molecular dynamics simulations, followed by MM/GBSA binding free energy calculations and per-residue decomposition analyses. Results: Docking results indicated that the majority of modeled P proteins were able to adopt energetically favorable interaction poses with H_2_N_2_F_2_, with predicted binding energies ranging from −3.7 to −7.2 kcal·mol^−1^. The most favorable docking energies were observed for GII.6_S9c_KC576910 (−7.2 kcal·mol^−1^), GII.3_MX_U22498 (−7.1 kcal·mol^−1^), and GII.4_CARGDS11182_OR700741 (−6.8 kcal·mol^−1^). Molecular dynamics simulations suggested stable ligand engagement within canonical HBGA-binding pockets, with recurrent residues such as Asp374, Gln393, and Arg345 contributing to electrostatic and hydrophobic interactions, consistent with previously reported HBGA-binding motifs. MM/GBSA analyses revealed comparatively favorable binding tendencies among these complexes, particularly for globally prevalent genotypes including GII.3, GII.4, and GII.6. Conclusions: This work provides a large-scale structural and energetic assessment of the potential interactions between a naturally occurring lettuce-derived fucosylated hexasaccharide and human norovirus P domains. The results support the notion that core-fucosylated food-associated glycans can serve as interaction partners for diverse norovirus genotypes and offer comparative molecular insights into glycan recognition patterns relevant to foodborne transmission. The integrative AlphaFold3–docking–dynamics framework presented here may facilitate future investigations of virus–glycan interactions within food matrices.

## 1. Introduction

Human noroviruses are major microbial contaminants responsible for widespread food safety incidents, accounting for more than 90% of non-bacterial gastroenteritis outbreaks worldwide [1,2]. Noroviruses belong to the genus Norovirus within the family *Caliciviridae*. Their genomes consist of a single-stranded, positive-sense RNA of approximately 7.5 kb. Except for murine norovirus, the genome typically contains three open reading frames (ORFs) [3]. Among these, ORF2 encodes the major structural protein VP1, which comprises the shell (S) domain that encapsulates the viral RNA and the protruding (P) domain responsible for receptor and antibody recognition. The P protein can be further divided into the P1 and P2 subdomains, where the exposed P2 subdomain forms the apex of the capsid arch and serves as the putative receptor-binding domain mediating host interaction and immune recognition [4,5]. Notably, Norovirus in ten genogroups (GI-GX) can be further divided into 48 confirmed capsid genotypes (9 GI, 26 GII, 3 GIII, 2 GIV, 2 GV, 2 GVI and 1 genotype each for GVII, GVIII, GIX [formerly GII.15] and GX) based on amino acids of the complete VP1 [6]. Genogroup GI, GII, GIV, GVIII, and GIX infect humans, with GII and GI predominance. The uncorrected pairwise distance ranges for strain, cluster, and genogroup were 0–14.1%, 14.3–43.8%, and 44.9–61.4%, respectively [7].

Accumulating evidence indicates that histo-blood group antigens (HBGAs) act as receptors or co-receptors for human noroviruses and that their interactions with viral capsid proteins are critical for viral attachment, infection, and transmission [8,9]. HBGAs are glycans characterized by specific oligosaccharide sequences that contain terminal fucosyl residues and typically occur as *N*- or *O*-linked glycans on glycoproteins or glycolipids [10]. These glycans are widely distributed on epithelial cells across various mammalian tissues and can also exist as free or conjugated oligosaccharides in mucosal secretions of the gastrointestinal, respiratory, and genitourinary tracts, as well as in body fluids such as blood, intestinal contents, and breast milk [11]. Volunteer challenge studies have demonstrated a strong correlation between host blood type and susceptibility to norovirus infection [12]. Individuals with blood type O are highly susceptible to GI.1 infection, whereas those with blood type B are largely resistant; non-secretor individuals also display complete resistance to GI.1 infection. Structural and phenotypic analyses have further revealed the glycan-binding profiles of uncommon genotypes such as GII.13 and GII.21, the epidemic recombinant GII.P16-GII.2, and the novel genotypes GII.23-25, elucidating their genotype-specific interactions with HBGAs [13]. Mutations in the P2 region have been repeatedly linked to altered HBGA-binding profiles, antigenic drift, and immune escape, especially among epidemic genotypes such as GII.4 [14]. Consequently, despite sharing a conserved capsid architecture, P domains from different genogroups and genotypes can differ markedly in their glycan recognition properties, underscoring the necessity of genotype-resolved structural modeling when assessing virus–glycan interactions [15].

Carbohydrate compounds also play a pivotal role in the foodborne transmission of noroviruses [16,17]. It is estimated that 14–23% of global norovirus infections result from contaminated food, with bivalve mollusks (e.g., oysters, mussels, and clams) and fresh or frozen produce acting as the main transmission vehicles. Current hypotheses regarding virus–food matrix interactions include specific recognition of carbohydrate-like molecules [18], non-specific adsorption [19], and internalization into plant tissues [20]. Distinct food types exhibit different binding capacities toward noroviruses, suggesting interaction specificity likely derived from the presence of diverse HBGA-like ligands in food matrices. Virus-like particles (VLPs) of GII.4 noroviruses have been shown to specifically recognize carbohydrate residues in lettuce cell wall extracts, particularly HBGA-like structures containing core fucosyl groups [9]. Similarly, Tian et al. demonstrated that oyster gastrointestinal tissues contain A-type HBGA-like substances and later identified H-type epitopes in other shellfish such as clams and mussels, confirming their role in facilitating norovirus adsorption [21].

Our research group has long focused on the molecular mechanisms underlying the pathogenicity and transmission of noroviruses. Using integrated multi-omics approaches combining glycomics, proteomics, and genomics, we previously identified a specific ligand in lettuce that can bind norovirus [22]. This ligand, a fucosylated hexasaccharide designated H_2_N_2_F_2_, consists of two hexoses, two *N*-acetylhexosamines, and two fucosyl residues. It contains antigenic determinants corresponding to type A, H, and Lewis a HBGAs and was shown to specifically associate with GII.4 norovirus P proteins in lettuce tissues. However, due to its structural complexity, both chemical and enzymatic synthesis of H_2_N_2_F_2_ remain challenging, precluding direct experimental verification of its binding behavior. Consequently, the precise molecular basis governing the interactions between such core-fucosylated foodborne hexasaccharide and norovirus capsid proteins remains unresolved.

The determination of viral receptor-binding protein structures is essential for understanding host recognition mechanisms [23]. Although several crystal structures of norovirus P domains in complex with histo-blood group antigens have been reported, these structures are restricted to a limited number of genotypes and canonical HBGA ligands, and therefore do not capture the full genetic and antigenic diversity of human noroviruses. Notably, no experimental complex structures are currently available for the structurally complex, food-derived core-fucosylated hexasaccharide H_2_N_2_F_2_ investigated in this study, which precludes reliable homology-based modeling of P-domain–glycan complexes. Recent advances in computational structural biology have provided powerful alternatives. Over the past decade, the advent of deep learning-based prediction models, notably the AlphaFold series, has revolutionized protein structure prediction, achieving near-experimental accuracy [24]. These models have enabled systematic exploration of protein folding, evolution, and receptor-binding mechanisms across viral genotypes. As such, integrative computational modeling has become indispensable for delineating viral receptor interactions, predicting host specificity, and informing antiviral design [25]. Under these constraints, molecular docking offers a practical and standardized strategy to systematically explore potential interaction modes and relative binding tendencies across a large and genetically diverse set of norovirus P proteins, while AlphaFold-based complex prediction for virus–glycan systems remains to be fully benchmarked.

In this study, we utilized AlphaFold3 to predict the three-dimensional structures of 182 norovirus P proteins, representing all known genotypes across five human norovirus genogroups (GI, GII, GIV, GVIII, and GIX). The predicted models were validated through stereochemical analysis and then subjected to systematic molecular docking against the core-fucosylated foodborne hexasaccharide H_2_N_2_F_2_. Representative P protein dimers with the strongest predicted affinities were further examined by molecular dynamics simulations and MM/GBSA energy decomposition to investigate the stability and detailed binding energetics of the complexes. By integrating high-accuracy structure prediction with computational interaction modeling, this work established a comprehensive structural framework to elucidate both conserved and genotype-specific recognition patterns between human noroviruses and core-fucosylated foodborne glycans. The findings provide molecular-level evidence linking food-derived glycans to norovirus attachment and persistence, offering new insights into the mechanisms of foodborne transmission and potential targets for preventive interventions.

## 2. Materials and Methods

### 2.1. Sequence Retrieval and Structure Prediction

A total of 182 complete VP1 gene reference sequences of human noroviruses were retrieved from the Human Calicivirus Typing Tool Database (https://calicivirustypingtool.cdc.gov/becerance.cgi, accessed on 27 May 2024) in FASTA format. PhyloSuite was used to conduct, manage, and streamline the analyses [26]. The dataset covered a broad genetic spectrum, including GI.1-9, GI.ancestral, GII.1-27, GII.ancestral, GIV.1, GIV.NA1, GVIII.1, and GIX.1, with at least two representative sequences for each genotype. For the GII.4 lineage, multiple epidemic variants such as the Den Haag, Sydney, and Hong Kong strains were included to capture intra-genotypic diversity.

Three-dimensional structural models of the P domains were predicted using AlphaFold3 (AF3) (https://alphafoldserver.com/, accessed on 27 June 2025) [27]. Structural validation was conducted by inspecting Ramachandran plots generated via PROCHECK to evaluate stereochemical quality. Models with more than 90% of residues in the most favored regions were considered geometrically acceptable for subsequent molecular docking. Model confidence was further evaluated using the per-residue predicted Local Distance Difference Test (pLDDT) scores provided by AlphaFold3. Particular attention was given to residues comprising the canonical HBGA-binding pocket within the P2 subdomain, in order to confirm that the predicted interaction interfaces were supported by high local confidence prior to molecular docking analyses.

### 2.2. Validation of the Docking Procedure

Because H_2_N_2_F_2_ is a structurally complex core-fucosylated hexasaccharide with high conformational flexibility, direct docking without prior benchmarking may introduce uncertainty in pose prediction. Therefore, before applying the docking protocol to H_2_N_2_F_2_, we first evaluated its reliability using previously resolved norovirus P protein–HBGA co-crystal structures. To establish the reliability of the docking protocol, previously resolved norovirus P protein–HBGA complex structures were obtained from the Protein Data Bank (PDB, https://www.rcsb.org/). Representative structures included GI.7 + H-type HBGA (4P1V), GII.21 + Lea (4RM0), GII.4 + Leb (4OPS), GI.7 + Lex (4P2N), GI.7 + Ley (4P25), GII.4 + B-type HBGA (5IYQ, 4X06), and GII.4 + Ley (4WZE).

For each complex, the co-crystallized HBGA ligand was separated from the P protein and redocked into the corresponding binding pocket using AutoDock Vina v1.1.2 [28]. The docking grid was centered on the P2 subdomain region as defined in the original crystal structures, with the exhaustiveness parameter set to 16. Root-Mean-Square-Deviation (RMSD) values between the redocked and crystallographic ligand poses were subsequently calculated to assess the consistency and pose reproducibility of the docking configuration.

### 2.3. Molecular Docking of H_2_N_2_F_2_ with Predicted P Proteins

After confirming the reliability of the docking protocol using experimentally resolved norovirus P protein–HBGA complexes, the validated procedure was subsequently applied to investigate the interactions between AF3-predicted P proteins and the foodborne hexasaccharide H_2_N_2_F_2_. Dimeric models of all AF3-predicted P proteins were generated based on the GII.4 dimeric template (PDB ID: 4WZE) using the PyMoL align module. The core-fucosylated foodborne hexasaccharide H_2_N_2_F_2_ was used as the docking ligand. The 2D structure of H_2_N_2_F_2_ was drawn by ChemBioDraw Ultra 14.0 and converted to 3D structure by ChemBio3D Ultra 14.0 software, which was further energy-minimized using the MM2 force field.

Docking simulations were performed using Autodock Vina v1.1.2. The docking grid was positioned to cover the HBGA-binding pocket on the P2 subdomain, with exhaustiveness set to 16. The lowest docking affinity (kcal/mol) was recorded for each protein–ligand pair. The top three complexes with the strongest binding affinities were selected for molecular dynamics simulations.

### 2.4. Molecular Dynamics Simulations

Molecular dynamics (MD) simulations were performed using Amber14 [29]. The compound H_2_N_2_F_2_ was first prepared by ACPYPE, a tool based on ANTECHAMBER for generating automatic topologies and parameters in different formats for different molecular mechanics programs, including the calculation of partial charges. Then, the forcefield “leaprc.gaff” (generalized amber forcefield) was used to prepare the ligand, while “leaprc.ff14SB” was used for the receptor. The system was placed in a rectangular box (with a 10.0 Å boundry) of TIP3P water using the “SolvateOct” command with the minimum distance between any solute atoms. Equilibration of the solvated complex was performed by carrying out a short minimization (5000 steps of each steepest descent and conjugate gradient method), 500 ps of heating, and 50 ps of density equilibration with weak restraints using the GPU (NVIDIA^®^ Tesla K20c) accelerated PMEMD (Particle Mesh Ewald Molecular Dynamics) module. Finally, 40 ns of the MD simulations were carried out. Trajectory analyses were performed with cpptraj to calculate RMSD and root-mean-square fluctuation (RMSF).

### 2.5. Binding Free Energy and Energy Decomposition per Residue Calculations

The binding free energies (ΔG_bind_ in kcal/mol) were calculated using the Molecular Mechanics/Generalized Born Surface Area (MM/GBSA) method, implemented in AmberTools [30]. Moreover, to identify the key protein residues responsible for the ligand binding process, the binding free energy was decomposed on a per-residue basis. For each complex, the binding free energy of MM/GBSA was estimated as follows:

ΔG_bind_ = G_complex_ − G_protein_ − G_ligand_

where ΔG_bind_ is the binding free energy and G_complex_, G_protein_ and G_ligand_ are the free energies of the complex, protein, and ligand, respectively.

It should be noted that MM/GBSA-derived binding free energy values are known to overestimate absolute binding strengths. In this study, MM/GBSA calculations were therefore employed primarily for comparative analysis of the relative binding tendencies among different genotypes and for identifying key residues contributing to ligand recognition.

## 3. Results

### 3.1. Structural Prediction of P Proteins

All 182 truncated P protein sequences were successfully modeled using AlphaFold3. The resulting structures showed the characteristic P1 and P2 subdomains consistent with known norovirus capsid topologies. Ramachandran analysis confirmed that all models possessed good stereochemical geometry, with more than 90% of residues located in the most favored regions. The structural diversity across genotypes was well-captured, especially among the multiple GII.4 variants, supporting the use of these models for comparative docking studies.

Analysis of AlphaFold3 confidence metrics showed that the mean pLDDT values across the 182 P protein models ranged from 82.85 to 88.40. As these values represent averages over the entire P domain, the moderately reduced scores primarily reflect increased conformational flexibility at the *N*- and *C*-terminal loop regions, which are known to be structurally dynamic and less constrained. In contrast, residues forming the canonical HBGA-binding pocket within the P2 subdomain consistently exhibited high pLDDT scores, indicating strong local structural confidence at the predicted ligand–binding interfaces. Together, these results support the suitability of the predicted P protein models for subsequent molecular docking and molecular dynamics analyses.

### 3.2. Validation of Docking Accuracy

**Figure 1:**
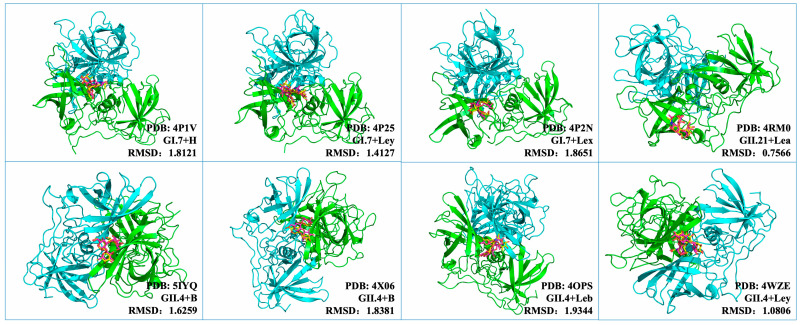
Validation of Docking Accuracy. The superimposed conformations of the redocked ligands (yellow) and the original crystallographic ligands (magenta) are shown for eight representative complexes. Each panel is annotated with the PDB ID, the corresponding norovirus genotype, the HBGA type, and the RMSD value (Å) between the redocked and crystallographic ligand poses.

To evaluate docking performance, native HBGA ligands were redocked into their respective crystal structures. The RMSD values between the redocked and crystallographic ligand poses were 0.757 Å for GII.21 + Lea (4RM0), 1.079 Å for GII.4 + Ley (4WZE), 1.409 Å for GI.7 + Ley (4P25), 1.805 Å for GII.4 + B-type HBGA (5IYQ), 1.812 Å for GI.7 + H-type HBGA (4P1V), 1.931 Å for GI.7 + Lex (4P2N), and 1.934 Å for GII.4 + Leb (4OPS) (Figure 1). These results demonstrated that the chosen docking parameters reproduced the experimental binding conformations with high accuracy. The dimeric form of the P protein consistently achieved lower RMSD values than the monomeric form, indicating improved stability of the docking model.

### 3.3. Docking Results of H_2_N_2_F_2_ with Predicted P Proteins

**Figure 2:**
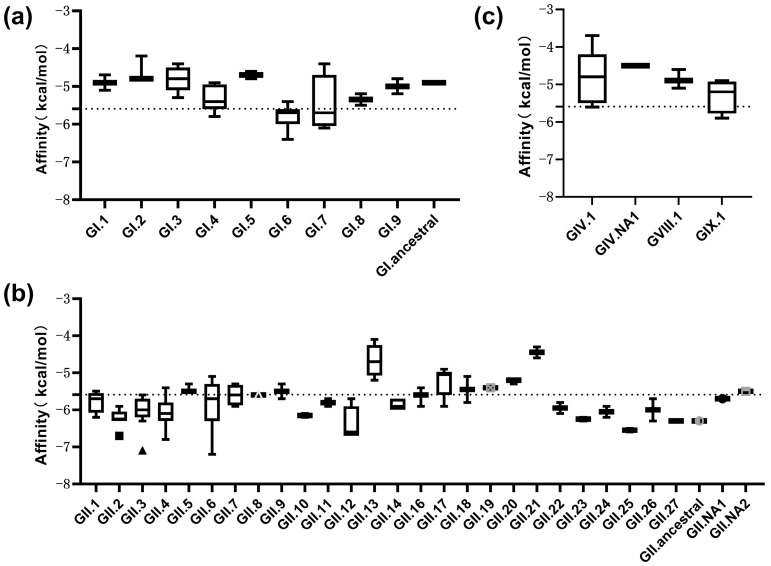
Docking of H_2_N_2_F_2_ with predicted P proteins. Boxplots of binding affinities (kcal·mol^−1^) for H_2_N_2_F_2_ docked with the (**a**) GI genogroup, (**b**) GII genogroup, and (**c**) other genogroups. Mean values are indicated by dashed lines.

Docking of the H_2_N_2_F_2_ ligand with the 182 modeled P protein dimers yielded predicted binding free energies ranging from −3.7 to −7.2 kcal·mol^−1^, with an overall mean of −5.59 kcal·mol^−1^ and a median of −5.70 kcal·mol^−1^. When comparing different genogroups, most predicted affinities of the GI-type genotypes (GI.1–GI.9 and GI.ancestral) were distributed between −4.5 and −6.0 kcal·mol^−1^ and exhibited an average predicted affinity of approximately −5.07 kcal·mol^−1^ (Figure 2a), whereas for the GII-type genotypes (GII.1–GII.27, GII.NA1, GII.NA2, and GII.ancestral), the predicted binding energies spanned a broader range, with several genotypes exhibiting values below −6.0 kcal·mol^−1^. The distribution of GII docking affinities extended toward lower energy values compared with GI genotypes with a comparatively lower mean docking energy of about −5.74 kcal·mol^−1^ (Figure 2b). Predicted affinities for genogroups GIV, GVIII, and GIX are shown in Figure 2c, with docking energies generally distributed between approximately −4.5 and −5.8 kcal·mol^−1^ across the analyzed genotypes.

Among all the modeled complexes, the three most energetically favorable docking poses were observed for GII.6_S9c_KC576910 (−7.2 kcal·mol^−1^), GII.3_MX_U22498 (−7.1 kcal·mol^−1^), and GII.4_CARGDS11182_OR700741 (−6.8 kcal·mol^−1^). These three proteins corresponded to genotypes GII.6, GII.3, and GII.4, which displayed the lowest predicted docking energies in this study. Consistently, the mean predicted affinities of the GII.3, GII.4, and GII.6 genotypes were approximately −6.01, −6.06, and −5.83 kcal·mol^−1^, respectively. The combined mean affinity of these three genotypes (−5.90 kcal·mol^−1^) was lower than the overall averages of both GI and GII genogroups, suggesting comparatively favorable interaction tendencies with H_2_N_2_F_2_.

### 3.4. Molecular Dynamics Simulation Results

**Figure 3:**
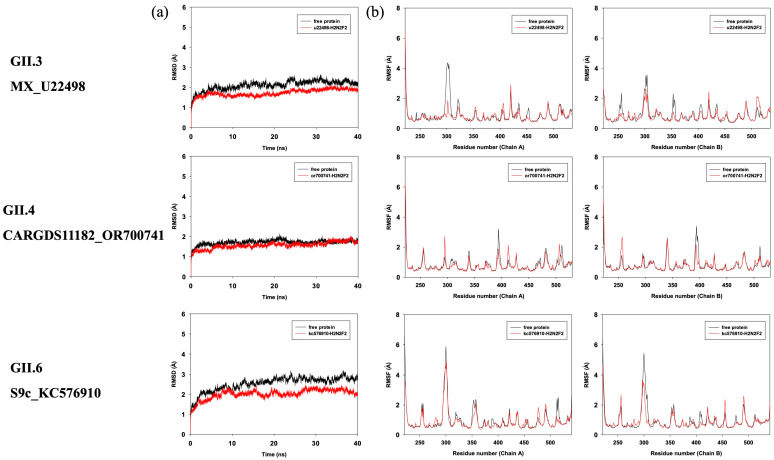
RMSD and RMSF analyses of the three top-ranked P protein–H_2_N_2_F_2_ complexes. (**a**) Root-mean-square deviations (RMSDs) of all atoms in each P protein–H_2_N_2_F_2_ complex relative to the initial structure as a function of simulation time. (**b**) Root-mean-square fluctuations (RMSFs) of residues in the P protein–H_2_N_2_F_2_ complexes and corresponding free P proteins during 40 ns simulations.

To explore the potential binding modes between H_2_N_2_F_2_ and the three top-ranked GII.6_S9c_KC576910-H_2_N_2_F_2_, GII.3_MX_U22498-H_2_N_2_F_2_, and GII.4_CARGDS11182_OR700741-H_2_N_2_F_2_, molecular dynamics (MD) simulations were performed using the Amber14 software package. The preferential binding mechanisms of these three P proteins with H_2_N_2_F_2_ were evaluated by 40 ns MD simulations based on the docking results. To assess the dynamic stability of the complexes and validate the sampling strategy, the root-mean-square deviation (RMSD) values of the protein backbones throughout the simulations were calculated and are shown in Figure 3a. As illustrated, the backbone RMSD values reached equilibrium, indicating that all three complexes remained conformationally stable over the simulation period.

The RMSF of all residues in both the free P proteins and the corresponding P protein–H_2_N_2_F_2_ complexes were also calculated to evaluate residue flexibility (Figure 3b). Distinct fluctuations were observed between the bound and unbound states, particularly around the binding pockets. Residues directly involved in the interaction with H_2_N_2_F_2_ exhibited reduced flexibility, with RMSF values generally below 2 Å, suggesting that ligand binding contributes to local structural stabilization within the binding site.

To further elucidate the contribution of individual residues to ligand binding, the electrostatic, Van der Waals, solvation, and total interaction energies were decomposed using the MM-GBSA method. The per-residue energy contributions were divided into Van der Waals (ΔE_vdw), solvation (ΔE_sol), electrostatic (ΔE_ele), and total energy (ΔE_total) terms.

**Figure 4:**
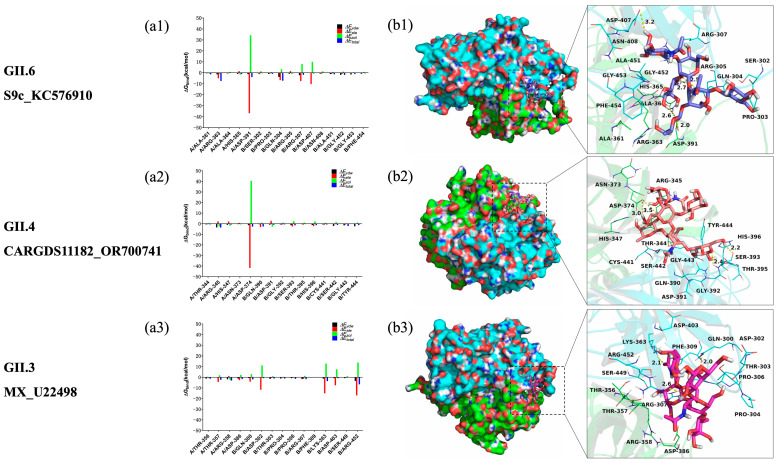
Binding modes and per-residue energy decomposition of the three top-ranked P protein–H_2_N_2_F_2_ complexes. (**a1**–**a3**) Per-residue decomposition of binding free energy for each P protein–H_2_N_2_F_2_ complex. (**b1**–**b3**) Predicted binding modes of H_2_N_2_F_2_ within the P protein binding pockets obtained from MD simulations. Chain A is shown in green and chain B in cyan, with hydrogen bonds represented by yellow dashed lines.

In the GII.6_S9c_KC576910–H_2_N_2_F_2_ complex, residue A/Asp-391 exhibited a strong electrostatic contribution (ΔE_ele < −36.0 kcal/mol) (Figure 4(a1)) and formed two hydrogen bonds with H_2_N_2_F_2_, with bond lengths of 2.0 Å and 2.6 Å (Figure 4(b1)). Residue B/Gln-304 also contributed electrostatically (ΔE_ele < −6.0 kcal/mol) and formed two additional hydrogen bonds (2.5 Å and 2.7 Å). The same residue showed a notable Van der Waals contribution (ΔE_vdw < −3.5 kcal/mol), indicating strong hydrophobic interactions with the ligand. Overall, Van der Waals interactions dominated the binding interface, mainly involving hydrophobic residues such as A/Ala-361, A/Ala-364, B/Pro-303, B/Ala-451, and B/Phe-454. The calculated total binding free energy (ΔG_bind) was −51.4 kcal/mol, indicating comparatively favorable interaction energetics between H_2_N_2_F_2_ and GII.6_S9c_KC576910.

In the GII.4_CARGDS11182_OR700741–H_2_N_2_F_2_ complex, residue A/Asp-374 provided the dominant electrostatic interaction (ΔE_ele < −40.0 kcal/mol) (Figure 4(a2)), forming two hydrogen bonds with H_2_N_2_F_2_ (bond lengths 3.0 Å and 3.5 Å; Figure 4(b2)). Additionally, residue A/Arg-345 exhibited a pronounced Van der Waals contribution (ΔE_vdw < −3.0 kcal/mol), reflecting close contact with the ligand. As in the previous complex, Van der Waals interactions were the main stabilizing forces. The total binding free energy (ΔG_bind = −30.3 kcal/mol) confirmed the ability of H_2_N_2_F_2_ to bind effectively to the GII.4 P protein.

For the GII.3_MX_U22498–H_2_N_2_F_2_ complex, residue B/Arg-452 displayed a strong electrostatic contribution (ΔE_ele < −17.0 kcal/mol) (Figure 4(a3)) and formed a hydrogen bond of 2.6 Å with the ligand (Figure 4(b3)). Residue B/Lys-363 also contributed significantly (ΔE_ele < −15.0 kcal/mol) through a hydrogen bond of 2.1 Å. Furthermore, B/Arg-452 exhibited a strong Van der Waals contribution (ΔE_vdw < −3.0 kcal/mol), reflecting close spatial interaction with H_2_N_2_F_2_. Van der Waals forces were again the predominant contributors to binding, primarily involving hydrophobic residues such as B/Pro-304, B/Pro-306, and B/Phe-309. The calculated ΔG_bind was −45.7 kcal/mol, indicating that H_2_N_2_F_2_ forms a stable and energetically favorable complex with the GII.3 P protein. 

The molecular dynamics simulations consistently demonstrated that H_2_N_2_F_2_ is dynamically stable under simulation conditions with the P proteins of genotypes GII.3, GII.4, and GII.6. Electrostatic forces contributed to initial ligand recognition, while Van der Waals and hydrophobic interactions played dominant roles in stabilizing the complexes. Per-residue energy decomposition analysis further revealed that ligand binding was primarily driven by a limited number of recurrent residues within the P2 subdomain. Across the analyzed complexes, residues such as Asp374, Gln393, Tyr389, and Arg345 consistently contributed favorable electrostatic energies, whereas residues including Leu345 and Val365 provided stabilizing Van der Waals interactions. The calculated MM/GBSA binding free energy values (−30.3 to −51.4 kcal/mol) indicated relatively favorable binding tendencies among the three selected complexes. Consistent with previous reports, these values were interpreted in a comparative manner to assess relative binding trends and residue-level contributions. Together with the molecular dynamics analyses, these results provide comparative structural insights into interaction patterns that may contribute to the observed binding specificity of the core-fucosylated foodborne hexasaccharide H_2_N_2_F_2_ toward the human norovirus P domain.

## 4. Discussion

This study provides a comprehensive structural and energetic characterization of the interactions between the core-fucosylated foodborne hexasaccharide H_2_N_2_F_2_ and the P domains of human noroviruses. By integrating AlphaFold3-based structural modeling, validated docking, and molecular dynamics simulations, we established a full-spectrum comparative framework encompassing all known human norovirus genotypes (overlapping GI, GII, GIV, GVIII, and GIX genogroups). This approach enabled a systematic examination of the predicted structural interactions between a naturally occurring plant oligosaccharide previously identified in lettuce and diverse human norovirus P proteins [22]. Given that H_2_N_2_F_2_ is structurally complex and remains difficult to synthesize, computational modeling represents a necessary and rational means to investigate its binding potential and molecular mechanism.

Docking analyses suggested that all modeled P proteins were able to adopt predicted interaction poses with H_2_N_2_F_2_ under the applied computational conditions, with calculated docking energies ranging from −3.7 to −7.2 kcal·mol^−1^. Among the 182 predicted structures, only three P proteins—from genotypes GI.4, GII.13, and GIV.1—did form recurrent hydrogen-bond interactions, with binding energies of −4.9, −4.7, and −4.2 kcal·mol^−1^, respectively. In contrast, other sequences within these same genotypes did form stable hydrogen bonds, suggesting that subtle amino acid substitutions within the P2 subdomain can markedly influence the binding pocket geometry and ligand accessibility. It should be noted that the absence or reduction in hydrogen bonds in a given docking pose does not preclude alternative or transient interaction modes, particularly given the conformational flexibility of both the P domain and the oligosaccharide ligand. Such microstructural variation may contribute to genotype-specific differences in receptor recognition, which have been implicated in host range diversity among human noroviruses. Given that the P protein of the norovirus capsid contains the receptor-binding domain, which governs host attachment and is prone to adaptive mutations [31], understanding the molecular determinants of its ligand recognition is essential for elucidating viral transmission and host specificity.

Comparative mapping of the binding interfaces revealed a conserved constellation of residues that govern ligand recognition. Aspartate, glutamine, and arginine residues contributed primarily to electrostatic stabilization, while alanine and proline residues mediated extensive Van der Waals and hydrophobic contacts, reflecting a cooperative balance between polar and nonpolar interactions. These features are consistent with crystallographic findings in GII.4 and related genotypes, where residues such as Asp374, Gln393, and Arg345 mediate hydrogen bonding and stacking with A-, H-, and Lewis-type HBGAs [32,33,34]. The recurrence of these residues in our simulations reinforces the structural authenticity of the predicted complexes and validates the computational framework.

The molecular dynamics simulations further indicated that H_2_N_2_F_2_ remained dynamically associated within the canonical HBGA-binding pockets of the three strongest complexes—GII.6_S9c_KC576910, GII.3_MX_U22498, and GII.4_CARGDS11182_OR700741—throughout the 40 ns trajectories. RMSF analyses revealed reduced flexibility of key binding-site residues, suggesting local conformational stabilization upon ligand engagement. Electrostatic interactions appeared to dominate the initial recognition process, whereas Van der Waals and hydrophobic interactions contributed primarily to the sustained stabilization of the complexes. Notably, among the tested 182 human norovirus P proteins, comparatively more favorable predicted binding tendencies were observed for GII.6, GII.3, and GII.4, which are also among the genotypes most frequently implicated in foodborne outbreaks worldwide [35,36]. It should be noted, however, that norovirus epidemiological dynamics are multifactorial and are influenced by a combination of immune escape, replication fitness, environmental stability, and host susceptibility. This alignment between relative binding tendencies derived from computational analyses and epidemiological prevalence suggests a potential association between glycan recognition properties and genotype circulation patterns.

It should be emphasized that the MM/GBSA binding free energy values reported here are not intended to represent absolute experimental affinities. Accordingly, the docking and molecular dynamics analyses presented here should be viewed as a comparative and hypothesis-generating framework. MM/GBSA calculations are known to systematically overestimate binding free energies, particularly for large and flexible ligands such as oligosaccharides. Accordingly, in this study, MM/GBSA results were used to compare relative binding tendencies across genotypes and elucidate residue-level energetic contributions within the predicted complexes. This comparative framework enables the assessment of conserved and genotype-dependent interaction features while remaining mindful of the inherent limitations of purely computational approaches.

Experimental validation of H_2_N_2_F_2_–norovirus interactions is currently constrained by practical considerations. The H_2_N_2_F_2_ hexasaccharide possesses a highly complex core-fucosylated structure that remains difficult to synthesize in vitro, and the isolation and purification of defined glycan components from food matrices such as lettuce present additional technical challenges. Under these constraints, computational docking and molecular dynamics simulations provide a feasible and informative strategy to explore potential interaction patterns and residue-level contributions. While the present study does not seek to establish definitive mechanistic conclusions, it offers a comparative structural framework that may help guide future experimental efforts as synthetic glycan chemistry and glycomics-based methodologies continue to advance.

Taken together, these findings extend previous observations that core-fucosylated or HBGA-like carbohydrates in food matrices can mediate norovirus attachment. Such glycans have been identified in oysters, leafy vegetables, and other food sources [16,21], where they may provide anchoring sites for viral particles. By structurally characterizing the interaction between an authentic lettuce-derived glycan and multiple human norovirus genotypes, the present study supports the notion that naturally occurring food-associated glycans may act as environmental binding partners, potentially influencing virus adherence and persistence in foodborne transmission contexts.

## 5. Conclusions

In summary, this study provides a systematic computational analysis of the potential interactions between human norovirus P proteins and a food-derived core-fucosylated hexasaccharide. The results indicate that multiple norovirus genotypes are capable of engaging H_2_N_2_F_2_ through a combination of electrostatic and hydrophobic interactions, suggesting conserved structural features involved in glycan recognition. Comparatively more favorable binding tendencies were observed for the globally prevalent GII.3, GII.4, and GII.6 genotypes, which may be associated with their ability to interact with core-fucosylated glycans, although norovirus epidemiological dynamics are known to be multifactorial. Together, these findings offer comparative molecular insights into genotype-dependent glycan recognition and provide a structural framework that may inform future experimental studies and the exploration of intervention strategies targeting virus–glycan interactions during food production and processing.

## Data Availability

Data are contained within the article and Supplementary Materials.

## Supplementary Materials

The following supporting information can be downloaded at: https://www.mdpi.com/article/10.3390/v18010131/s1, Table S1: Structural confidence metrics and docking affinity values for the 182 predicted human norovirus P proteins.

## Abbreviations

The following abbreviations are used in this manuscript:

ORFs	Open reading frames
HBGAs	Histo-blood group antigens
VLPs	Virus-like particles
MD	Molecular dynamics
RMSD	Root-mean-square deviation
RMSF	Root-mean-square fluctuation
