# Supplementary material for: Structural Insights into the Interaction Between a Core-Fucosylated Foodborne Hexasaccharide (H2N2F2) and Human Norovirus P Proteins
Source: Viruses. 2026 Jan 20;18(1):131. doi: 10.3390/v18010131 (PMC12846348; doi:10.3390/v18010131)
Supplement: Supplementary file 1 [file viruses-18-00131-s001.zip › viruses-4041229-supplementary.pdf]

Protein name	ncbi ID	genotype
GII.6_S9c_KC576910	KC576910	GII.6
GII.3_MX_U22498	U22498	GII.3
GII.4_CARGDS11182_OR700741	OR700741	GII.4
GII.12_SaitamaU1_AB039775	AB039775	GII.12
GII.12_StGeorge_GQ845370	GQ845370	GII.12
GII.2_SnowMountain_AY134748	AY134748	GII.2
GII.25_Dhaka1928_MG495083	MG495083	GII.25
GII.4_Osaka_Osaka_AB434770	AB434770	GII.4
GII.4_Sydney_WI467121_KX354134	KX354134	GII.4
GII.12_PunePC24_EU921353	EU921353	GII.12
GII.25_Beijing53931_GQ856469	GQ856469	GII.25
GII.4_Yerseke_Isumi060936_AB294790	AB294790	GII.4
GI.6_No20Saitama9817_LC342057	LC342057	GI.6
GII.4_Den_Haag_DenHaag_EF126965	EF126965	GII.4
GII.4_Sydney_Sydney_JX459908	JX459908	GII.4
GII.4_Sydney_Sydney2105Y1677_OL336352	OL336352	GII.4
GII.6_30443_KM198534	KM198534	GII.6
GII.2_HenrytonSP17_MF405169	MF405169	GII.2
GII.2_MK04_DQ456824	DQ456824	GII.2
GII.2_OsakaNI_DQ366347	DQ366347	GII.2
GII.23_Loreto6422_MG495080	MG495080	GII.23
GII.26_Leipzig07788a_MF352142	MF352142	GII.26
GII.27_Arg15813_MK733205	MK733205	GII.27
GII.27_Loreto0959_MG495077	MG495077	GII.27
GII.3_RotterdamP1D0_AB385626	AB385626	GII.3
GII.4_Asia_Asia_AB220921	AB220921	GII.4
GII.4_New_Orleans_NSW001P_GQ845367	GQ845367	GII.4
GII.4_Sydney_OH16002_LC153121	LC153121	GII.4
GII.ancestral_Tokyo684_AB684728	AB684728	GII.ancestr
GII.1_Dillingen391_AF425767	AF425767	GII.1
GII.10_Erfurt546_AF427118	AF427118	GII.10
GII.2_SantaRosa1764_KY865306	KY865306	GII.2
GII.2_Vaals_AB281090	AB281090	GII.2
GII.23_Loreto1847_KT290889	KT290889	GII.23
GII.24_Loreto1972_KY225989	KY225989	GII.24
GII.3_RotterdamE1300327_MF140689	MF140689	GII.3
GII.4_Asia_Sakai04179_AB220922	AB220922	GII.4
GII.4_Farmington_Hills_Langen1061_AY485642	AY485642	GII.4
GII.4_Osaka_PunePC51_EU921388	EU921388	GII.4
GII.4_Sydney_WoononaNSW3309_JX459907	JX459907	GII.4
GII.6_GZ2010L96Guangzhou_JX989075	JX989075	GII.6
GI.7_IF2036_AY675555	AY675555	GI.7
GII.10_Mc37_AY237415	AY237415	GII.10
GII.22_Dhaka1940_MG495082	MG495082	GII.22
GII.3_HK71_JX846924	JX846924	GII.3
GII.4_Botswana1026_MW019618	MW019618	GII.4
GII.4_Farmington_Hills_FarmingtonHills_AY502023	AY502023	GII.4
GI.6_Beijing55063_GQ856464	GQ856464	GI.6
GI.7_Dhaka1882_MH130046	MH130046	GI.7

GII.14_Beijing55028_GQ856465	GQ856465	GII.14
GII.14_SendaiYG99_KJ196278	KJ196278	GII.14
GII.2_Melksham_X81879	X81879	GII.2
GII.3_Amsterdam1_KJ194500	KJ194500	GII.3
GII.3_Arg320_AF190817	AF190817	GII.3
GII.4_Allegany0230_MT028542	MT028542	GII.4
GII.4_Hunter_Hunter504D04O_DQ078814	DQ078814	GII.4
GI.6_VA497_AF538678	AF538678	GI.6
GII.11_Sw918_AB074893	AB074893	GII.11
GII.14_Maizuru8533_GU017903	GU017903	GII.14
GII.16_Tiffin_AY502010	AY502010	GII.16
GII.17_Kawasaki323_AB983218	AB983218	GII.17
GII.2_KL109_JX846925	JX846925	GII.2
GII.24_EdenPrairie5457_MG495084	MG495084	GII.24
GII.4_Den_Haag_Shimane2_AB541348	AB541348	GII.4
GII.4_Sydney_B7265_MW521126	MW521126	GII.4
GII.4_Yerseke_Yerseke_EF126963	EF126963	GII.4
GII.7_Gwynedd273_AF414409	AF414409	GII.7
GIX.1_SapporoHK299_KJ196290	KJ196290	GIX.1
GI.4_Chiba407_AB042808	AB042808	GI.4
GII.18_OHQW125_AY823305	AY823305	GII.18
GII.22_Yuri_AB083780	AB083780	GII.22
GII.3_109_MH218579	MH218579	GII.3
GII.4_CHDC2094_FJ537135	FJ537135	GII.4
GII.4_Den_Haag_Minerva_EU078417	EU078417	GII.4
GII.4_Hong_Kong_CUHKNS2200_MN400355	MN400355	GII.4
GII.7_236_MH218692	MH218692	GII.7
GI.6_Beijing53997_GQ856463	GQ856463	GI.6
GI.6_BS5(Hesse)_AF093797	AF093797	GI.6
GI.7_Chiba030100_AJ844469	AJ844469	GI.7
GII.1_Ascension208_JN797508	JN797508	GII.1
GII.1_Hawaii_U07611	U07611	GII.1
GII.11_Sw43_AB126320	AB126320	GII.11
GII.12_No35Saitama991_LC342059	LC342059	GII.12
GII.14_M7_AY130761	AY130761	GII.14
GII.14_Saga8610_GU594162	GU594162	GII.14
GII.26_Leon4509_KU306738	KU306738	GII.26
GII.3_Herzberg385_AF539439	AF539439	GII.3
GII.3_SN2000JA_AB190457	AB190457	GII.3
GII.4_CHDC5191_FJ537134	FJ537134	GII.4
GII.4_Hunter_Hunter_AY883096	AY883096	GII.4
GII.4_US9596_US9596_AF414424	AF414424	GII.4
GII.6_1455_KX158281	KX158281	GII.6
GII.6_Miami292_AF414410	AF414410	GII.6
GII.9_VA97207_AY038599	AY038599	GII.9
GII.NA1_Loreto1257_MG495079	MG495079	GII.NA1
GI.6_WUG1_AB081723	AB081723	GI.6
GII.16_Neustrelitz260_AY772730	AY772730	GII.16
GII.3_Milwaukee009_JN565063	JN565063	GII.3
GII.3_Toronto_U02030	U02030	GII.3

GII.4_New_Orleans_Hokkaido3_AB933767	AB933767	GII.4
GII.4_San_Francisco_SanFranciscoSA_OR262344	OR262344	GII.4
GII.6_SaitamaU4_AB039777	AB039777	GII.6
GII.8_SaitamaU25_AB039780	AB039780	GII.8
GIV.1_OC01017023_LC101824	LC101824	GIV.1
GI.8_Boxer_AF538679	AF538679	GI.8
GII.1_Amsterdam3_KJ194507	KJ194507	GII.1
GII.17_Kawasaki308_LC037415	LC037415	GII.17
GII.4_San_Francisco_SanFranciscoUSA_OR262322	OR262322	GII.4
GII.5_SaitamaT49_KJ196277	KJ196277	GII.5
GII.5_SaitamaT52_KJ196288	KJ196288	GII.5
GII.6_TCHE9913646_GU930737	GU930737	GII.6
GII.NA2_PNV06929_MG706448	MG706448	GII.NA2
GIV.1_LakeMacquarie_JQ613567	JQ613567	GIV.1
GIV.1_SaintCloud_AF414427	AF414427	GIV.1
GI.4_Beijing55169_GQ856475	GQ856475	GI.4
GI.4_Koblenz433_AF394960	AF394960	GI.4
GI.6_14BC1_KP027330	KP027330	GI.6
GII.16_Hiram_AY502006	AY502006	GII.16
GII.19_OHQW170_AY823306	AY823306	GII.19
GII.4_New_Orleans_NewOrleans_GU445325	GU445325	GII.4
GII.7_Leeds_AJ277608	AJ277608	GII.7
GIX.1_Mex7076_AF542090	AF542090	GIX.1
GI.3_DesertShield395_U04469	U04469	GI.3
GII.20_Luckewalde591_EU373815	EU373815	GII.20
GII.5_Hillingdon_AJ277607	AJ277607	GII.5
GII.7_TAKAsanKimchi_KJ196295	KJ196295	GII.7
GII.9_GoulburnValleyG5175C_DQ379715	DQ379715	GII.9
GI.8_NagoyaKY531_KJ196298	KJ196298	GI.8
GI.9_Vancouver730_HQ637267	HQ637267	GI.9
GII.13_Fayetteville_AY113106	AY113106	GII.13
GII.20_Leverkusen267_EU424333	EU424333	GII.20
GII.20_OC071182007_AB542917	AB542917	GII.20
GI.1_Norwalk_M87661	M87661	GI.1
GI.3_Beijing55042_GQ856473	GQ856473	GI.3
GI.3_Potsdam196_AF439267	AF439267	GI.3
GII.17_CUHKNS682_KT589391	KT589391	GII.17
GII.18_OHQW101_AY823304	AY823304	GII.18
GII.6_SaitamaU16_AB039778	AB039778	GII.6
GII.6_Shizuoka8913_HM633213	HM633213	GII.6
GIV.1_Alph23_DQ093067	DQ093067	GIV.1
GVIII.1_Tokyo218_AB985418	AB985418	GVIII.1
GI.3_ShimizuKK2866_KJ196292	KJ196292	GI.3
GI.4_Groningen_LN854563	LN854563	GI.4
GI.7_Providence_JN899243	JN899243	GI.7
GII.17_CSE1_AY502009	AY502009	GII.17
GII.17_SaitamaT87_KJ196286	KJ196286	GII.17
GIX.1_Beijing55161_GQ856474	GQ856474	GIX.1
GI.1_Gothenburg_EU085529	EU085529	GI.1
GI.3_Beijing54114_GQ856471	GQ856471	GI.3

GI.4_Queensarmsleeds92_AJ313030	AJ313030	GI.4
GI.ancestral_Tokyo455_AB684696	AB684696	GI.ancestra
GII.17_Katrina17_DQ438972	DQ438972	GII.17
GIX.1_J23_AY130762	AY130762	GIX.1
GVIII.1_Chiba040502_AJ844470	AJ844470	GVIII.1
GI.2_Constellation59_AF435807	AF435807	GI.2
GI.2_Southampton_L07418	L07418	GI.2
GI.3_Beijing54108_GQ856470	GQ856470	GI.3
GI.3_Otofuke_AB187514	AB187514	GI.3
GI.5_OC020180_LC101825	LC101825	GI.5
GI.9_CAIQ12110628_KF586507	KF586507	GI.9
GIV.1_CanetenRoussillon_KT030674	KT030674	GIV.1
GI.1_SRSVKY8989J_L23828	L23828	GI.1
GI.3_Beijing54660_GQ856472	GQ856472	GI.3
GI.5_AppalachicolaBay318_AF414406	AF414406	GI.5
GI.5_YiwuCB1_MH443711	MH443711	GI.5
GII.13_PunePC25_EU921354	EU921354	GII.13
GII.13_SaitamaT80_KJ196276	KJ196276	GII.13
GI.5_SzUG1_AB039774	AB039774	GI.5
GII.21_KawasakiYO284_KJ196284	KJ196284	GII.21
GVIII.1_YuzawaGira2HS_KJ196291	KJ196291	GVIII.1
GI.3_Stavanger_AF145709	AF145709	GI.3
GI.3_VA98115_AY038598	AY038598	GI.3
GIV.NA1_WI7002_KX907728	KX907728	GIV.NA1
GI.3_OS32_LC122713	LC122713	GI.3
GI.7_AlbertaEI404_KU311161	KU311161	GI.7
GII.21_IF1998_AY675554	AY675554	GII.21
GI.2_Leuven_FJ515294	FJ515294	GI.2
GIV.1_Ahrenshoop246_JQ970479	JQ970479	GIV.1
GIV.1_CCDCGR111359_KC894731	KC894731	GIV.1
GIV.1_FortLauderdale_AF414426	AF414426	GIV.1
GII.13_GoulburnValleyG5175B_DQ379714	DQ379714	GII.13
GIV.1_Italy980_FM865412	FM865412	GIV.1

Ramachandran Plot	Affinity（kcal/mol）	Hydrogen bond
90.0	-7.2	Yes
90.0	-7.1	Yes
90.2	-6.8	Yes
91.1	-6.7	Yes
90.3	-6.7	Yes
91.0	-6.7	Yes
90.7	-6.6	Yes
90.5	-6.6	Yes
90.2	-6.6	Yes
90.3	-6.5	Yes
91.1	-6.5	Yes
91.4	-6.5	Yes
90.8	-6.4	Yes
90.8	-6.4	Yes
91.3	-6.4	Yes
90.2	-6.4	Yes
91.3	-6.4	Yes
90.6	-6.3	Yes
91.0	-6.3	Yes
91.4	-6.3	Yes
91.7	-6.3	Yes
90.6	-6.3	Yes
90.2	-6.3	Yes
90.9	-6.3	Yes
90.4	-6.3	Yes
90.8	-6.3	Yes
90.6	-6.3	Yes
90.6	-6.3	Yes
92.0	-6.3	Yes
90.7	-6.2	Yes
91.1	-6.2	Yes
90.6	-6.2	Yes
90.2	-6.2	Yes
90.5	-6.2	Yes
90.1	-6.2	Yes
90.4	-6.2	Yes
90.5	-6.2	Yes
90.0	-6.2	Yes
90.5	-6.2	Yes
90.6	-6.2	Yes
91.2	-6.2	Yes
91.4	-6.1	Yes
90.7	-6.1	Yes
90.4	-6.1	Yes
90.8	-6.1	Yes
90.5	-6.1	Yes
90.8	-6.1	Yes
91.7	-6.0	Yes
91.5	-6.0	Yes

91.9	-6.0	Yes
91.7	-6.0	Yes
91.0	-6.0	Yes
91.1	-6.0	Yes
91.2	-6.0	Yes
91.1	-6.0	Yes
91.3	-6.0	Yes
93.7	-5.9	Yes
90.3	-5.9	Yes
90.9	-5.9	Yes
90.1	-5.9	Yes
90.1	-5.9	Yes
91.4	-5.9	Yes
90.5	-5.9	Yes
90.9	-5.9	Yes
91.3	-5.9	Yes
90.5	-5.9	Yes
90.5	-5.9	Yes
90.6	-5.9	Yes
90.9	-5.8	Yes
88.7	-5.8	Yes
90.0	-5.8	Yes
90.7	-5.8	Yes
90.5	-5.8	Yes
90.1	-5.8	Yes
90.7	-5.8	Yes
90.7	-5.8	Yes
92.9	-5.7	Yes
93.3	-5.7	Yes
90.7	-5.7	Yes
91.0	-5.7	Yes
90.6	-5.7	Yes
90.3	-5.7	Yes
91.1	-5.7	Yes
91.3	-5.7	Yes
90.6	-5.7	Yes
90.5	-5.7	Yes
90.3	-5.7	Yes
91.1	-5.7	Yes
92.4	-5.7	Yes
91.1	-5.7	Yes
90.5	-5.7	Yes
89.2	-5.7	Yes
90.8	-5.7	Yes
90.7	-5.7	Yes
90.8	-5.7	Yes
90.9	-5.6	Yes
90.8	-5.6	Yes
90.0	-5.6	Yes
90.4	-5.6	Yes

90.1	-5.6	Yes
91.4	-5.6	Yes
90.5	-5.6	Yes
92.1	-5.6	Yes
92.0	-5.6	Yes
92.7	-5.5	Yes
92.2	-5.5	Yes
90.3	-5.5	Yes
91.3	-5.5	Yes
91.0	-5.5	Yes
90.6	-5.5	Yes
90.4	-5.5	Yes
90.1	-5.5	Yes
91.0	-5.5	Yes
91.3	-5.5	Yes
92.2	-5.4	Yes
90.5	-5.4	Yes
92.5	-5.4	Yes
90.5	-5.4	Yes
90.3	-5.4	Yes
90.1	-5.4	Yes
90.6	-5.4	Yes
91.1	-5.4	Yes
90.0	-5.3	Yes
91.9	-5.3	Yes
91.0	-5.3	Yes
91.1	-5.3	Yes
90.7	-5.3	Yes
92.7	-5.2	Yes
92.1	-5.2	Yes
90.3	-5.2	Yes
90.6	-5.2	Yes
90.6	-5.2	Yes
92.4	-5.1	Yes
90.8	-5.1	Yes
92.2	-5.1	Yes
91.0	-5.1	Yes
90.5	-5.1	Yes
90.7	-5.1	Yes
91.5	-5.1	Yes
91.3	-5.1	Yes
91.8	-5.1	Yes
93.0	-5.0	Yes
90.9	-5.0	Yes
90.9	-5.0	Yes
91.1	-5.0	Yes
91.1	-5.0	Yes
91.8	-5.0	Yes
90.3	-4.9	Yes
92.6	-4.9	Yes

91.3	-4.9	No
92.7	-4.9	Yes
91.8	-4.9	Yes
91.4	-4.9	Yes
91.7	-4.9	Yes
90.9	-4.8	Yes
92.1	-4.8	Yes
91.0	-4.8	Yes
91.8	-4.8	Yes
91.9	-4.8	Yes
92.4	-4.8	Yes
92.1	-4.8	Yes
92.0	-4.7	Yes
92.2	-4.7	Yes
91.4	-4.7	Yes
90.3	-4.7	Yes
90.7	-4.7	Yes
90.8	-4.7	No
90.0	-4.6	Yes
91.2	-4.6	Yes
92.1	-4.6	Yes
93.4	-4.5	Yes
91.8	-4.5	Yes
91.9	-4.5	Yes
90.6	-4.4	Yes
90.5	-4.4	Yes
90.3	-4.3	Yes
90.6	-4.2	Yes
92.4	-4.2	Yes
91.3	-4.2	Yes
90.6	-4.2	No
91.2	-4.1	Yes
92.4	-3.7	Yes
